# Pediculated Accessory Liver Lobe with Gallbladder in a Preterm with Umbilical Cord Hernia

**DOI:** 10.3390/children9111754

**Published:** 2022-11-15

**Authors:** Martha Georgina Brandtner, Hannah N. Stundner-Ladenhauf, Sara Lapointe-Rohde, Christa Schimke, Dietrich Kluth, Roman Metzger

**Affiliations:** 1 Department of Pediatric and Adolescent Surgery, Paracelsus Medical University Salzburg, 5020 Salzburg, Austria; 2 Department of Visceral, Transplant and Thoracic Surgery, Innsbruck Medical University, 6020 Innsbruck, Austria; 3 Department of Oral and Maxillofacial Surgery, Klinikum Wels-Grieskirchen, 4600 Wels, Austria; 4 Department of Pediatric Surgery, University of Leipzig, 04109 Leipzig, Germany

**Keywords:** umbilical cord hernia, abdominal wall defect, accessory liver lobe, children

## Abstract

(1) Background: Accessory liver lobes are a rare finding and only a few case reports of accessory liver lobes in abdominal wall defects have been reported so far. In the case of a congenital wall defect including liver parenchyma, there is still an ongoing debate on the definition of the abdominal wall defect and best care practice. Even though congenital abdominal wall defects are frequently diagnosed in prenatal screenings, controversy on the underlying etiology, embryology and underlying anatomy remains. Prenatal distinction between omphalocele and hernia into the cord cannot always be obtained; however, due to its clinical relevance for postnatal management and counseling of parents, accurate diagnosis is essential. (2) Case Presentation: We describe the uncommon postnatal finding of a pediculated accessory liver lobe with gallbladder in a preterm with umbilical cord hernia, which was prenatally diagnosed as omphalocele. Postnatal examination revealed an amniotic sac with a diameter of six and a small abdominal wall defect of three centimeters in diameter. Postnatal management included resection of the accessory liver lobe and gallbladder and closure of the defect. (3) Results and (4) Conclusions: Throughout the literature, the distinction between umbilical cord hernia and omphalocele has been variable. This has led to confusion and difficulties regarding postnatal treatment options. In order to achieve an accurate prenatal and/or postnatal diagnosis, the morphological differences and clinical manifestation of umbilical cord hernia and omphalocele need to be assessed. Further embryological studies are warranted to understand the underlying embryological pathology of omphalocele and umbilical cord hernia and offer appropriate treatment. In consideration of possibly severe complications in the case of the torsion of a pedunculated accessory liver lobe, we strongly recommend primary removal once pre- or intraoperative identification has been made.

## 1. Introduction

Accessory liver lobes (ALL) are a rare finding and have mostly been encountered as incidental findings in adulthood, during abdominal surgery or autopsy [1,2]. In rare cases, an accessory liver lobe can even cause acute or recurrent abdomnial pain or impair liver function [3,4].

While the anatomic variations of liver vessels and bile ducts are common, ALLs are extremely rare and present as an extranumeric liver lobe composed of physiologically intact liver parenchyma. Due to the rare encounter of this anatomic variation, they are hardly considered in the differential diagnosis of uncommon hepatic findings. However, clinical episodes of vascular damage to the ALL [4] or the obstruction and compression of adjacent structure, such as the portal vein or stomach, have been reported [5]. In cases of pedunculated ALLs, torsion has been reported and may lead to symptoms, thus resulting in the diagnosis of the ALL [6]. Furthermore, ALLs can mimic tumors or, in rare cases, the hepatic parenchyma of the ALL itself can reveal signs of malignancy, such as hepatocellular carcinoma [7]. In some pediatric cases presented in the literature, diagnosis has been associated with an abdominal wall defect, such as omphalocele or umbilical cord hernia (UCH) [1,2,8].

In order to be able to distinguish abdominal wall defects, the definition of these two separate entities needs to be evaluated.

Currently, an umbilical cord hernia is defined as an abdominal wall defect with a diameter smaller than 4 cm, containing nothing but intestinal loops. On the contrary, in omphaloceles, the defect of the abdominal wall must measure over 4 cm to be defined as an omphalocele. In the case of an omphalocele, the amniotic sac may contain intestinal loops along with other visceral organs, such as the stomach or liver [9]. Unfortunately, the term omphalocele is also used as a hyperonym for UCH [2,10,11]. While one may argue that these definitions are only important on paper and not relevant to the patient, the distinction of these two entities is crucial. Other than omphaloceles, umbilical cord hernias are not considered to derive from a lateral fold defect and are therefore, according to the literature, not to be associated with cardiac or chromosomal anomalies, as seen in patients with omphaloceles [12]. Therefore, expectant parents with a prenatal finding of an umbilical cord hernia may not be counseled on the possibility of further diagnostics or genetic testing. However, in case of prenatal diagnosis of an omphalocele, genetic counseling and the discussion of postnatal surgical treatment options will be part of the routine prenatal workup in most countries. Precise prenatal differentiation between UCH and omphalocele is therefore essential, as incorrect diagnosis will alter prenatal management and/or cause unwarranted anxiety for parents [13]. 

## 2. Case Presentation

**Figure 1:**
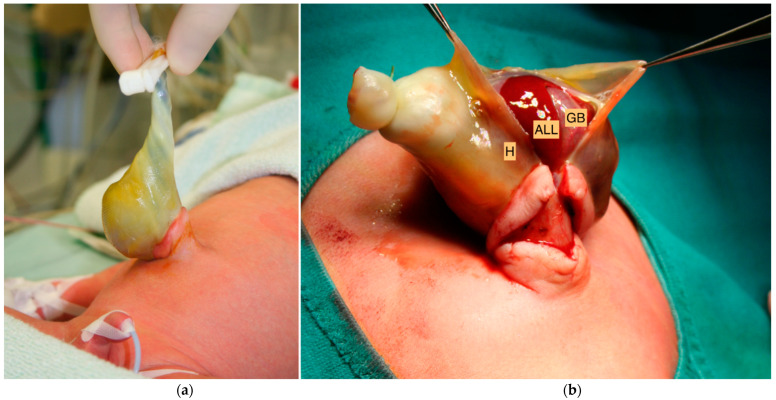
(**a**) A small abdominal wall defect of three centimeters in diameter and an intact hernia sac (H) were identified after birth. (**b**) Exposure of the accessory liver lobe (ALL) with embedded gallbladder (GB) after the opening of the membrane of the hernia sac (H).

We report a case of a male newborn with the prenatal diagnosis of a small abdominal wall defect. On prenatal ultrasounds, the herniation of the liver into the defect was suspected; therefore, parents were counseled on postnatal management. The patient was delivered at 35 + 6 gestational weeks with a birth weight of 2750 g by cesarean section due to placenta accreta. Postnatal examination showed a small abdominal wall defect of three centimeters in diameter and an intact amniotic sac with a protuberance above the umbilical ring measuring six centimeters (Figure 1a and 1b), including herniated liver parenchyma. Due to the size of the abdominal wall defect, the primary care pediatric surgeon diagnosed an umbilical cord hernia. However, since parts of the liver herniated into the sac, the correct identification of the defect according to the current literature was questionable. Aside from the UCH, perinatal hemodynamic adaption was uneventful. Postnatal examination revealed a small muscular (trabecular) ventricular septal defect, which was hemodynamically irrelevant, as well as a small persistent foramen ovale (PFO) and a persistent ductus arteriosus (PDA).

**Figure 2:**
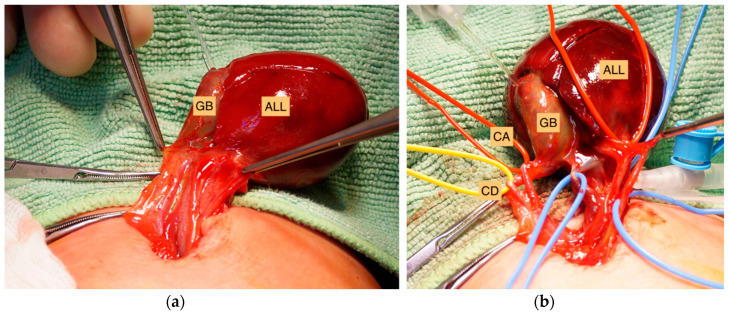
(**a**) Identification of the pediculated accessory liver lobe (ALL) with embedded gallbladder (GB). The ALL, cleaved into two liver segments, was completely separated from the abdominal liver and connected via a six-centimeter pedicle. (**b**) After the dissection of the pedicle of the ALL, eight structures containing blood vessels, a cystic duct (CD) and cystic artery (CA) were identified within the pedicle.

Since the patient was hemodynamically stable and there were no signs of the alteration of blood flow in the liver parenchyma, and surgical treatment was scheduled for the following day. On postnatal day one, the child underwent surgical exploration and the primary repair of the UCH. We identified separated but intact liver tissue with an embedded gallbladder separated from the abdominal liver and connected via a six-centimeter pedicle containing blood vessels and biliary ducts. The pedunculated liver tissue was adherent to the amniotic sac, and we identified at least two cleaved liver segments, most likely liver representing segments V and VI with an embedded gallbladder. Primarily, the ALL was relocated into the abdomen and the abdominal wall was closed to allow time for further diagnostic imaging. Sonography identified a normal portal vein and hepatic artery. The ALL was identified in the left lower quadrant of the abdomen. Bowel malrotation was excluded. After thorough investigation of the literature, it was decided to remove the ALL in order to prevent the torsion of the pediculated ALL, as described in the literature. An intraoperative cholangiography identified a very long cystic duct draining into a normal common bile duct. The exploration of the pedicle revealed at least eight vascular structures including the cystic artery and duct (Figure 2a and 2b). The clamping of all structures altered neither intraoperative blood flow nor the bile drainage of the liver. The ALL and gallbladder were safely removed. Histology revealed regular liver tissue and an intact gallbladder with signs of blood congestion. Recovery and follow up after four years were uneventful. 

## 3. Results and Discussion

### 3.1. Case Presentation

This case report presents us with two separate challenges. First, the definition and distinction between and umbilical cord hernia and an omphalocele, and secondly, the evaluation of the optimal management of a pedunculated accessory liver lobe in pediatric patients.

#### 3.1.1. Differentiation between Umbilical Cord Hernia and Omphalocele

Throughout the literature, UCH and omphalocele have not been separated as two different embryologic entities. This has led to confusion and difficulties regarding treatment options. Recent publications describe UCH as being associated with a higher mortality, cardiac anomalies and neural tube defects [11], which are true for omphaloceles but not UCH, by definition. Both entities derive from different defects that occur during gestation. This explains why chromosome analysis in UCH is without pathologic findings [13]. However, when counseling parents during prenatal exams on abdominal wall defects, the differentiation between UCH and omphalocele need to be considered. 

In order to obtain an accurate prenatal and/or postnatal diagnosis, the morphological differences and clinical manifestation of UCH and omphalocele need to be distinguished. 

Several studies claim that UCH solely affects the midgut and not visceral organs, such as the liver [10,12], therefore claiming that any abdominal wall defect containing visceral organs should be labelled an omphalocele. Similar to our case, Festen et al. claimed that UCH may contain liver parts aside from the midgut; however, it should be noted that by their definition, UCH accounts for a variation of omphalocele. They presented two cases of UCH, which appear to be nearly identical to our case: both contained a pediculated accessory liver lobe and gallbladder [2]. From review of the literature, the site of insertion of the umbilical cord and the attachment of the rectus muscles (xiphoid, costal margin) are estimated to be the best way to differentiate omphalocele from UCH [12,14,15]. Therefore, we recommend that the content of the amniotic sac should not be the term-defining criterion and we labelled the abdominal wall defect in our case as an UCH. 

However, whether the defect is determined to be an UCH or an omphalocele, in cases of the herniation of visceral organs, such as the liver, into the amniotic sac, we recommend immediate surgical exploration in order to prevent the strangulation of liver tissue, which may lead to ischemia and possible subsequent injury to the otherwise intact liver parenchyma and biliary tract. 

#### 3.1.2. Management of Accessory Liver Lobes

Accessory liver lobes are a rare anatomic variant. They present as a supranumeric liver lobe of intact hepatic parenchyma. While accessory liver lobes have some form of continuity with the original liver, ectopic liver lobes are completely separated and can be found in various locations [7]. Regarding the management of accessory liver lobes, there seems to be an ongoing debate whether to surgically treat these patients. In most cases, an ALL will be an accidental finding during some form of radiological imaging or during abdomnial surgery. Rare cases of liver function impairment, abdominal pain, bleeding, obstruction of the portal vein or malignancy have been reported [3,4,7,16]. If patients are found to be asymptomatic and without underlying conditions, such as hepatic disease, liver cirrhosis or malignancy, surgical treatment should not be obligatory. However, if adjacent structures or liver function are impaired or the patient presents with acute or recurrent abdominal pain or complications due to the size or location of the ALL, open or preferably laparoscopic resection should be discussed [7]. In cases of a large ALL or a pedunculated ALL, torsion has been reported and may result in the acute ischemia of the ALL or even the complete ischemia of the liver in case of large ALLs. In 2004, Ladurner et al. reported a severe case of hepatic ischemia due to the torsion of a large ALL, warranting a liver transplantation [17]. 

In pediatric patients, several reports of herniated liver parenchyma in regard to abdominal defects, such as umbilical cord hernias, can be found in the literature. They describe their findings as accessory liver lobes with an attached gallbladder that were only connected via vessels and bile ducts to the liver [2,8]. To our knowledge, the only pediatric ectopic liver with no connection to the intraabdominal liver was described by Fock et al. in 1963 [18].

Regarding the management of the herniation of liver parenchyma into the abdominal wall defect, the repositioning of the herniated liver parenchyma into the abdomen [11,19,20] seemed to be the option of choice. 

However, Elmasalme et al. reported a life threatening event of the torsion of an accessory live lobe in a six month old child, which was initially treated as a presumed pneumonia [8]. The child had previously undergone the primary closure of an omphalocele with the repositioning of an accessory liver lobe into the abdominal cavity. Only upon the second surgical consultation did the finding of an abdominal mass lead to the correct diagnosis of torsion [8]. Nagano et al. reported on a five year old child that presented with recurrent abdominal pain and vomiting [6]. Radiological imaging revealed a nonvascular lesion with the mixed echogenicity suspicious of an abscess. After antibiotic treatment, elective exploratory laparoscopy and subsequent resection was scheduled, which revealed an ALL with ischemic change due to possible torsion [6]. 

Goor et al. intended to classify the anomalies of the biliary tract depending on the biliary drainage from the lobe into the normal biliary tree and on the presence of a common capsule [21]. Three different types of accessory liver lobes were identified. In type I, a separate accessory lobe duct drains into an intrahepatic bile duct, while type II is defined by drainage into an extrahepatic bile duct. In type III, the accessory lobe presents with a common capsule with the normal liver. Our case would identify as type I, since a very long cystic duct drained into a normal intrahepatic common bile duct. According to Elmalsalme et al. this type may be at a higher risk for torsion due to the elongated pedicle [8]. Therefore, surgical removal is indicated in these types of accessory liver lobes. Further, Azmy et al. presented a case of the torsion of the gallbladder embedded in an accessory lobe of liver in a neonate with Beckwith–Wiedemann syndrome, requiring a second procedure for removal similar to the case presented in this report [1]. This highlights the importance of surgical excision on primary encounter of the ALL in order to prevent the child from further surgical treatment. 

To our knowledge, there is no case in the current literature reporting of complications during or after the removal of a pediculated accessory liver tissue. In consideration of the possibly severe complications in case of torsion as mentioned above, we strongly recommend primary removal of a pedunculated accessory liver lobe once pre- or intraoperative identification has been made.

## 4. Conclusions

Cases of UCH with accessory liver lobes connected via a pedicle to the abdominal liver are extremely rare. Further embryological studies are warranted in order to explore and understand the underlying embryological pathology of omphalocele and umbilical chord hernia. This is essential to properly distinguish both entities and to understand the development of accessory liver lobe formation.

The correct prenatal diagnosis of umbilical cord hernia should be aspired to allow adequately counseling of parents and to decide on prenatal management. One should be aware of the possibility of pediculated accessory liver lobes, and these should actively be searched for and surgically removed in order to prevent torsion and subsequent hepatic ischemia and spare the child from additional unnecessary procedures. The presence of an accessory liver lobe should be taken into consideration in children with a history of an omphalocele or umbilical cord hernia closure.

## Data Availability

Not applicable.
